# Cultural Sets Shape Adult Conceptualizations and Relationships to Nature

**DOI:** 10.3390/su132011266

**Published:** 2021-10-13

**Authors:** Linda Powers Tomasso, Jose Guillermo Cedeño Laurent, Jarvis T. Chen, Paul J. Catalano, John D. Spengler

**Affiliations:** 1 Department of Environmental Health, Harvard T.H. Chan School of Public Health, Boston, MA 02115, USA; 2 Population Health Sciences, Harvard University, Boston, MA 02115, USA; 3 Department of Social and Behavioral Sciences, Harvard T.H. Chan School of Public Health, Boston, MA 02115, USA; 4 Department of Data Science, Dana-Farber Cancer Institute, Boston, MA 02115, USA; 5 Department of Biostatistics, Harvard T.H. Chan School of Public Health, Boston, MA 02115, USA

**Keywords:** nature–health relationships, human–nature experience, urban nature, biophilia, nature exposure, focus group discussions, qualitative, photo research

## Abstract

The variability of nature and the nature construct have complicated interpretations of empirical evidence from nature-based health studies. The challenge of defining nature exposure for purposes of methodological standardization may encompass constructs beyond vegetated landcover. This study offers a new construct for defining ‘nature exposure’ that considers cultural sets and nature familiarity. Focus group discussions across the United States (N = 126) explored the concept of what constitutes the relationship to nature. The participant diversity included regions, cultural demographics, cumulative nature experience, and everyday nature exposure. Mixed methods of semi-structured discussion and a photo exercise that prompted nature connectedness allowed for data triangulation and the detection of contradictions between approaches. Individuals conceptualized nature in ways reflecting highly personal and differentiated experiences, which defied consensus toward a single nature construct. The group scoring of photo imagery showed consistent high and low levels of nature connectedness with respect to wildness and outdoor urban venues, respectively, but diverged in the assessment of nature within the built environment. Everyday nature exposure significantly differentiated how groups conceptualized and related to nature imagery. This result may indicate an unmet biophilic need among groups with low backgrounds of nature exposure. The contrasts between the discussion content and the observed reactions to nature imagery showed the value of using mixed methods in qualitative research.

## 1. Introduction

### 1.1. Background

The current understanding of associations between nature and health has relied on crude indicators of exposure to nature. Further, the benefits derived from exposure to nature are likely to vary depending on one’s innate connectedness to nature, which is nurtured by acquired experiences and stimulated biophilic responses [1]. Many studies on nature and wellbeing have acknowledged the role of personal affinity to nature [2–7]. Fewer considered how limited contact with nature [8–10] or health advisories proscribing outdoor play in polluted environments [11–13] lead to fear of or aversion toward nature. Demographic features of urban landscape contexts also influence wellbeing [14–17], although evidence is growing for how race, ethnicity, socioeconomic status (SES), gender, age, region, and urbanicity can modify nature relationships [18–23]. Attention to differences in the sociocultural underpinnings of contact with nature across diverse populations is highly germane to research on health and health disparities. The origins of nature relationships deserve scrutiny, as they combine with familiarity with nature and formative experiences to define a learned cultural set regarding nature. Opportunities to pursue comprehensive, inclusive ethnographic research on nature use by cultural sets are infrequent, however, such that knowledge of the bases, permanence, and plasticity of individual relationships to nature is incomplete [24]. Knowing if and where perceptual differences exist with respect to nature is a preliminary step for investigating why individuals and their identifying groups can vary in their relationships with, responses to, and use of nature-based environments.

### 1.2. Literature Review

Humans have evolved by relying on nature for existential, spiritual, and material fulfillment. Interacting with nature as a tangible rather than abstract premise has not made humans’ relationship with nature easier to apprehend. On one hand, natural structures have inspired biomimetic invention in fields as imaginative as poetry, aviation, and architecture. Conversely, immaterial concepts, such as sublimity and awe, are commonly illustrated by natural phenomena, such as sunsets and spider web geometry. Faced with the task of stating what ‘nature’ is, the Ionians chose only to assign nature a relational meaning, not to define it. Early philosophers identified “nature with some particular and definite thing and thus [gave] it a concrete definition. Thales said it was ‘water.’ Anaximenes said it was ‘air.’ Heraclitus said it was ‘fire’” [25]. Though the Greeks found it impossible to define the abstraction of nature, they nonetheless agreed on explaining its cosmogenesis and related that account to human wellbeing.

The literature supports the observation that human responses to “nature” are heterogeneous and quite varied. An early range of response evidence was centered on aesthetic preference and emotional experiences in nature rooted in environmental psychology [26–29]. Cultural factors entered research on urban nature responses, particularly for urban forests [30–34], thus strengthening the relevance of cultural preference for urban planning and design. Much has been learned through subsequent comparisons of physical, participatory, and affective responses to nature exposure according to sociodemographic groupings. In addition to race and ethnicity, studies have considered responses to nature according to urban and rural residency [35,36], Latino heritage [37,38], race, age, and residence [39–41], immigrant status [42–44], and disability [45], finding characteristics of these varied cultural settings that shape group-level nature relationships. However, the studies on each group of interest are few.

One of the strongest theoretical explanations for connection to nature is the biophilia hypothesis, which states that individuals have an intrinsic affinity for nature [46,47]. Biophilia sustains the desire for nature connectedness (NC) [48–50] and its close constructs of “nature relatedness“(NR) [51–54] and “nature affinity” [55,56]. These concepts strongly signal individuals’ predisposition for comfort in and valuation of nature [57]. Relating to nature contributes to health and wellbeing through anxiety reduction [58,59] and perceived restorativeness, which is linked to the biophilic quality of in situ natural environments [60]. For this study, we chose NC as our construct of interest to aid in the analysis of human–nature response patterns. NC emphasizes the cognitive component of nature associations and modes in which built environments impact nature relationships [61], which is consistent with the scoring instrument that this study will employ. Numerous validated scales developed to assess NC or NR [49,53,62–67] have been used in research to predict pro-environmental attitudes and their complementary behaviors [55,68–71] and to support the conclusion that environmental behaviors are strengthened by time in nature [2,72–77], which often has roots in childhood exposure and affinities cultivated during this formative imprinting [78–80].

Cultural sets may lead individuals to view and connect with nature differently. Sociodemographic examinations of NC have highlighted the influence of race [81–83], SES [84], and cultural politics [85] in evaluating nature participation. Race-based preference research generally considers urban parks and amenities to understand nature use among Blacks [86,87] and Hispanics [32] vis-a-vis whites, which is a shortcoming given the long trajectory of outdoor landscape use and environmental engagement by Black Americans [88,89]. Recent scholarship has shown that race does not differentiate white from non-white attitudes toward non-urban wildness among college students [81], despite exclusionary social attitudes and management protocols in wilderness, which may curtail Black use of those spaces [82,85,90]. SES differences impact the availability and quality of nature in urban greenspace in ways that are independent of race [91]. While linked to race, income and educational attainment predict opportunities for outdoor recreation [92,93], and low SES predicts that urban environmental risks deter outdoor play among poorer children [94–96]. Moreover, white attitudes toward nature are not monolithic and are shown to vary by gender [97,98] and age [99].

Viewing photo imagery as a nature exposure proxy follows previous study designs that use nature facsimiles to test their effects [100–102]. Simulating live nature exposure through photos has allowed researchers to test recovery from mental fatigue [103,104], examine psychological restoration [105–107], and measure subjective physical and mental energy in response to scenes of natural and built environments [108]. Photo use has also captured the variability in respondents’ perceptions of nature according to immigrant culture [109], nature preference by race/ethnicity [110,111], and correlation between aesthetic judgements and affective responses in appraising nature [112]. Kaplan and Talbot elaborated a three-phase photo presentation to discern preferences for built versus natural settings by race and identifiable variables underlying choice [34]. Photos of nature and built environments are methodologically useful as community audit tools [113–115], while participatory photo research has mapped positive emotional responses from encountering nature in everyday surroundings [116] and a “geo-narrative” of nature immersion [117].

The use of pictorial images as a research instrument has been recognized as an effective device for communicating a nuanced understanding for both individual and collective interpretations of nature [113,118]. From a pragmatic perspective, substituting imagery for live landscapes can reduce the logistical burdens of exposure comparisons and open to otherwise unattainable sociodemographic participation. Criticism of early environmental research for presenting exaggerated contrasts between natured vs. non-natured settings, e.g., inanimate urban vs. idyllic wilderness settings [102,119,120], or subjective, scenic quality assessments between older, white, mid-high SES respondents and younger, Black, low-SES respondents [114] has yielded more graduated comparisons, e.g., rural–urban settings [121,122]. Research has shown viewing both simulated and live nature to be restorative, though the greater effect was shown by live nature [123], while looking at nature imagery elicited the same psychological and physical effects of an actual nature encounter [124,125].

### 1.3. Hypothesis and Reasons for This Study

We pursue two objectives in this paper. First, we assess how individuals conceptualize nature and how cultural sets, which include nature familiarity, influence those appraisals. Second, we analyze how self-perceived NC influences biophilic interpretations of nature imagery in an effort to uncover any prototypical group-level similarities and distinctions among study participants that characterize nature relationships. We use nature imagery to test the hypothesis that varying background levels of nature will lead individuals to define and regard nature differentially according to their cultural sets, nature expectations, and urbanicity gradients—distinct interactions that may hold implications for human biophilic responses to contact with nature.

## 2. Materials and Study Design

### 2.1. Study Design and Methodology

This study combined qualitative and quantitative research methods [126–130]. Parallel mixed methods were used to examine instances of convergence and divergence between two data sources to examine individuals’ interactions with conceptually and pictorially expressed nature [131,132]. We compared coded qualitative data from semi-structured focus group discussions against quantitative data derived from NC scoring in the photo exercise to satisfy this approach. Mixed methods have been used in studies of nature, built environments, and health [101,133], though few public health studies were identified that utilized focus group discussion content supported by photo prompts to elicit participants’ opinions [134–136], as done here.

### 2.2. Study Recruitment

A two-stage recruitment process took place through Facebook advertisements placed in four U.S. metropolitan areas. The inclusion criteria included an adult age of 18+ and residence in one of the targeted metropolitan areas. A total of 596 participants registered through the online portal where they listed only city and gender, resulting in a metapopulation of 82.8% women, 15.0% men, and 1.5% gender non-conforming. All enrollees who identified as male or non-binary received emailed invitations to attend a focus group; females were invited chronologically until twelve participants per group confirmed attendance. Expanded recruitment through academic and community partnerships led to additional focus groups being held in targeted cities to generate a final study population (N = 126). Seventy-five individuals enrolled through Harvard Qualtrics (Facebook, Georgia State, Arizona State Universities), and 51 enrolled through outside organizational contacts (government conservation officials, land trust members, a community youth mentorship group, Latin American childcare givers). All final study participants voluntarily enrolled and provided electronic consent. Table S1 (Supplementary Materials) describes the focus group characteristics.

### 2.3. Study Sites

We convened participants in the environs of San Francisco, Atlanta, Phoenix, Boston, and Hartford for sessions at a community meeting room or university classroom. Exceptions were the session organized for conservation officers at their annual national conference and a final discussion over Zoom in accordance with IRB protocol restrictions due to COVID-19. Figure S1 illustrates the focus group locations.

### 2.4. Participatory Research Tools

**Figure 1.**
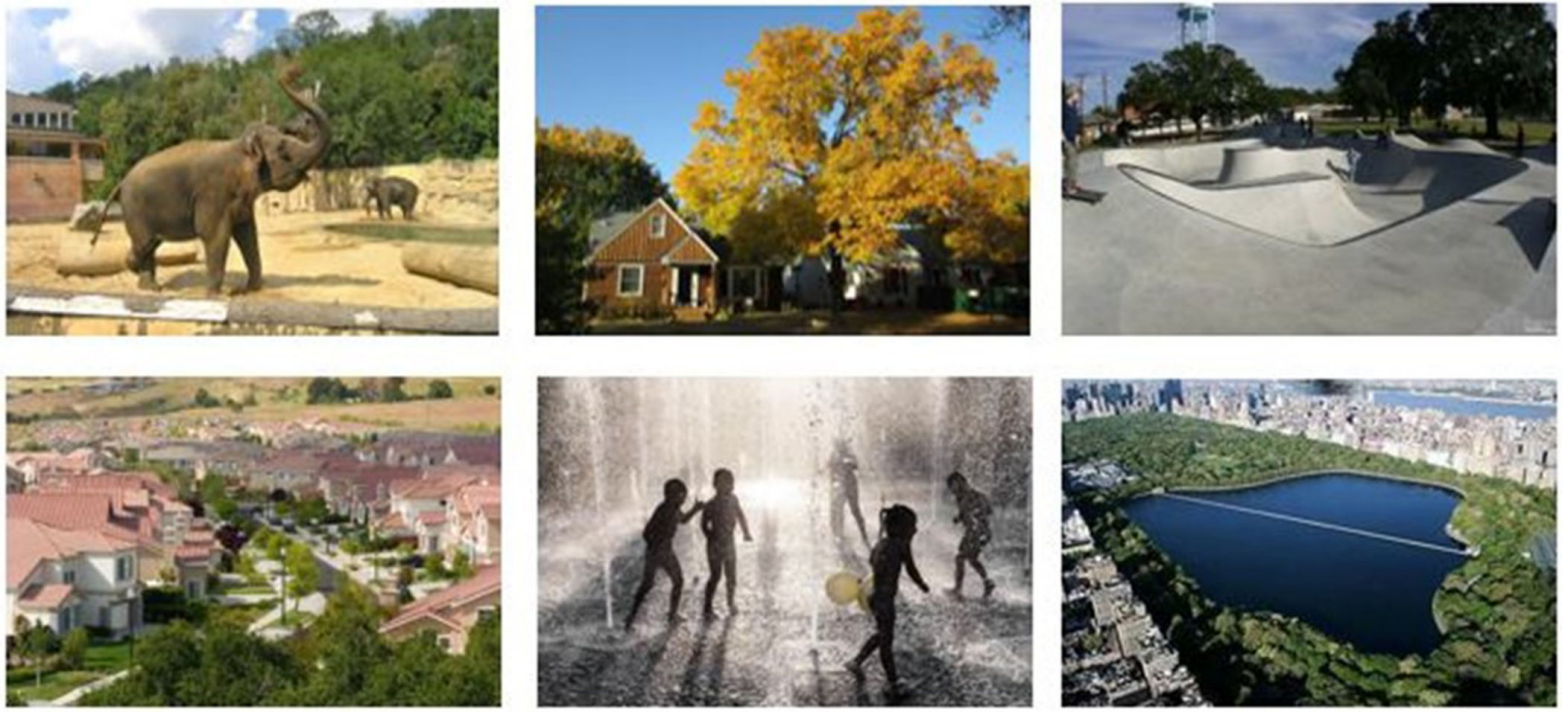
Sample photo images representing nature-based scenes to rate NC. The full photo array is shown in Figure S2.

The focus group sessions lasted 1.45 h and were divided into a one-hour semi-structured discussion and a 45-min group exercise in order to examine conventional nature-based scenes for the evocation of NC. The discussions conformed to a topic guide (Table S2) to solicit individual attitudes toward nature and were developed from background literature [137–140] and recommendations for further nature–health inquiries [24]. Two approximately equally sized subgroups were formed for the photo exercise; where possible, previously acquainted participants were separated to minimize correlated results. To operationalize the nature construct, the research team selected thirty-four photos with varying compositions of nature, human, and constructed objects for their potential to stimulate cross-cutting and interactive discussion based on universal scene familiarity and content objectivity [135]. Pictorial images ranged from biophilic elements within built environments to settings for outdoor activities and full nature landscapes (Figures 1 and S2). The exploration of the settings where most individuals might interact with nature gave license for our diverse participant base to describe any positive and negative affects experienced while viewing these scenes and to justify their intuitive feelings and concerns arising from the features depicted [118]. Each subgroup evaluated all 34 photos for the NC perceptions.

**Figure 2.**
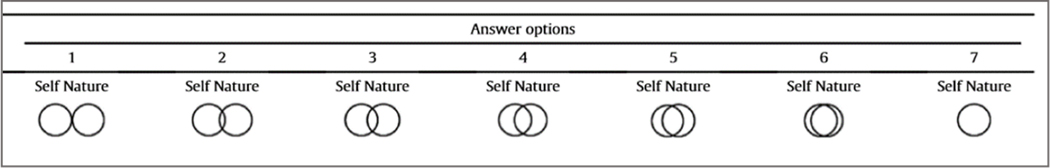
Inclusion of Nature in the Self scale (Schultz, 2002), a single-item scale measuring NC.

NC was self-assessed through the Inclusion of Nature in the Self (INS) scale, a validated, one-item psychometric tool [61]. The INS scale features seven degrees of NC that are indicated by overlapping Venn diagrams, where 1 represents exclusion of nature from the self and 7 represents oneness with nature (Figure 2). Participants were instructed to deliberate within their subgroups on the question “How connected to nature does this image make you feel?” as elicited prima facie by each photo and to explain their personal responses. The scoring resulted from a consensus process arrived at through group discussion and captured rationale for NC that would have been lost through silent scoring by individuals. The group deliberation around NC thus generated additional qualitative data that supplemented the semi-structured discussion around nature contact. One consensus-derived INS score was recorded on the paper strip shown in Figure 2 and affixed to each image. Outlier scores were also recorded if they fell two or more INS levels from the group consensus and were weighted in the final analysis. The researcher remained silent during photo discussions to allow the participants to freely interpret visual content without the interjection of language cues or researcher bias. The photo exercise produced 646 discrete NC scores for the photo set across the ten groups.

### 2.5. Data Collection

Data were collected during in-person focus group sessions held between September 2019 and January 2020 and over Zoom in November 2020. The interviews were digitally recorded with participants’ permission and transcribed verbatim to ensure accuracy. The transcripts contained no identifiable participant information. The subgroup photo discussions were audio-recorded simultaneously on two separate devices with the participants’ consent. Theoretical data saturation was determined when no new themes arose through on-going data analysis or the addition of diverse groups such that adequate sampling representation had been achieved [141]. Participants received a small retail gift card at the end of the session to acknowledge their time.

### 2.6. Data Analysis

Data analysis methods are briefly described here but are fully presented in Tomasso et al. [142]. Transcribed discussion content was analyzed and managed through a process of inductive thematic coding using the NVivo 12 Plus software [143]. Qualitative analysis of 163 transcript pages yielded 789 coded elements, 66 substantive themes, 15 organizational themes, and 3 synthesized final themes. After the initial transcript review, the first analyst identified codes based on prior subject knowledge and interfacing with the focus groups. She subsequently developed a code manual to define subthemes—specifying when and when not to use each [144]—to share with the second analyst. Each analyst independently coded the transcripts, with the second analyst identifying additional subthemes that produced a final codebook. Internal analytical validity was strengthened through the analysts’ regular discussions of contextual meaning through increasing familiarity with the transcripts’ contents and participants’ intentions, and open coding ascribed additional subthemes to the initial organizational scheme when omissions and new ideas were detected [130]. Inter-rater reliability was maintained through multiple and separate readings of transcripts to reconcile coding discrepancies; overlapping subthemes were merged and some nascent themes were dropped when the text evidence proved insufficient [145]. The two analysts reached concordance on 82.6% of the separately coded transcript content [127,146]. An unweighted kappa coefficient of 78.3% measured the analyst agreement and was corrected for chance.

The quotes in Tables 2–4 represent thematic data extracted from the coded transcripts and exemplify their respective organizational themes, e.g., defining nature, experiencing nature, and nature vis-à-vis the outdoors. INS scores from the photo exercise were categorized according to group-level nature exposure. INS scores from the photo exercise were categorized according to group-level nature exposure as determined by the density of surrounding built and natural environments of the focus group areas and participants’ comments. Categorical means for each nature scene were analyzed and visualized as ggplot2 boxplots. The normality of the data distribution was examined by means of the Shapiro–Wilk test, and the data were found to be non-normally distributed (W = 0.92, *p* < 2.2 × 10^−16^). Kruskal–Wallis rank sum tests were used to show significance in the differences in the mean INS scores among the three categories and as pair-wise group tests.

All statistical analyses were performed in R version 3.6.3. The Harvard T.H. Chan School of Public Health Institutional Review Board determined the study to be exempt under IRB reference number 19–1419 on 28 August 2019. Arizona and Georgia State Universities additionally consented to allow data collection under the referenced IRB.

## 3. Results

### 3.1. Focus Group Discussion

The first part of the focus group—where a set of participants that was diverse in terms of age, gender, geographic location, socioeconomic status, and race freely discussed their nature relationships—exposed a range of views based on cultural sets and nature familiarity. Still, three homogeneous populations emerged around levels of nature encountered in everyday living and working environments, which may have influenced perceived nature connectivity. We categorized the levels of nature exposure according to the participants’ comments about the frequency and opportunity of accessing nature and the density of the built environment of the focus group location (Table 1), hypothesizing that everyday exposure would condition biophilic inclinations toward nature, as depicted in the photos. We subsequently observed through the scoring consensus process that group-level nature exposure indeed shaped enthusiasms and degrees of connectedness toward the various nature images, and that categorization by everyday nature exposure translated into distinguishable NC patterns by group type.

Discussions showed that the cultural set and nature familiarity influenced response patterns in how individuals related to nature. Thematic analysis of the discussion transcripts revealed that participants had such divergent experiential reference points in nature that formed their own nature relationships that they prevented agreement on a shared nature construct. The comments in Table 2 reflect the subjectivity and diversity of nature experiences that participants felt had precluded agreement on a common nature construct within their respective groups. This disagreement speaks to the multiple meanings that participants attributed to individual nature experiences given the background nature familiarity, affinity, and opportunities in nature that they brought to the discussion.

Disagreement about the role and importance that individuals attributed to nature between focus groups was less pronounced within the focus groups. The cultural set, urbanicity, and level of everyday nature exposure distinctly influenced individuals’ positions toward nature. Individuals with lower nature exposure described their nature interactions more ecumenically, with repeated references to stars and the sky, weather, and insects. More nature-immersed individuals instead selectively qualified nature drawn from a variety of landscape-scale experiences. This divergence resulted in an imbalanced construct of nature based on lived experience, where those with less prior nature exposure tended toward a more expansive view, while those more highly immersed in nature favored the landscape scale and intensity of experiences while eliminating urban spaces from their definition of nature (Table 3).

Most individuals, irrespective of the group, disagreed with the idea that the outdoors and nature represent the same concept. In certain situations, the outdoors offered some consistent emotional benefits of time in nature, particularly when the outdoors was a familiar extension of one’s home, such as an outdoor patio or backyard garden. Urban individuals were more likely to reference the outdoors in terms of infrastructure, transit dependency, and schedules—factors that impede a sense of retreat that is available in purely nature-centered environments. A few participants described their desire to escape into nature even during moments outdoors (Table 4).

### 3.2. Photo Exercise for Rating Nature Connectedness

**Figure 3.**
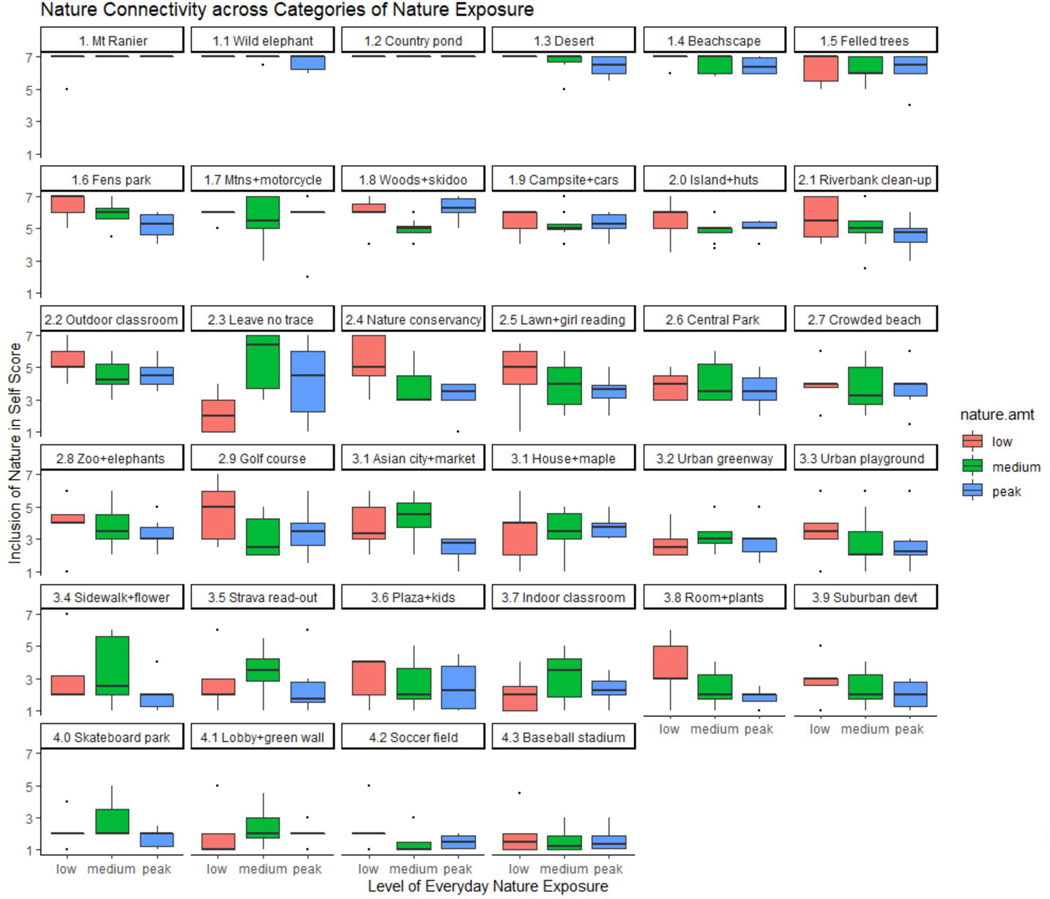
Nature photos were rated for NC by using the Inclusion of Nature in Self (INS) scale. The ratings are plotted by descending mean INS scores and by the categorical level. Focus groups were categorized by everyday nature exposure—low (red), medium (green), and peak (blue)—determined by the density of surrounding built and natural environments of the focus group areas and the participants’ comments.

The results of the photo exercise gave a more nuanced interpretation of how groups related to nature than the discussion comments alone. The photos allowed the focus groups to operationalize the construct of NC by using the INS scale across a range of familiar nature scenes. The mean INS scores for each photo image were grouped categorically by everyday nature exposure categories: Peak-level nature exposure characterized conservation wardens, land conservationists, and Western-based graduate students (blue, N = 204); medium-level exposure characterized the Facebook ad respondents in Boston, Berkeley, suburban Atlanta, and Phoenix (green, N = 272); low-level exposure characterized the participants in urban Atlanta and urban Connecticut and the Latin American childcare workers in Sacramento (red, N = 170). The results are depicted in Figure 3.

Despite the great diversity in lifetime nature engagement within the focus groups, general agreement was observed between categorical levels of everyday nature exposure and INS scores to signal what does and does not constitute nature. All three groups registered low mean scores for scenes that did not inspire NC and similarly high means for scenes that were indisputably natured. However, for images depicting nature found within the built environment, the INS scores trended higher among groups from nature-deprived areas and lower among groups who were more accustomed to abundant nature. Groups with medium-level nature exposure often arrived at higher mean NC scores and larger scoring ranges than either the peak- or low-exposure groups.

**Figure 4.**
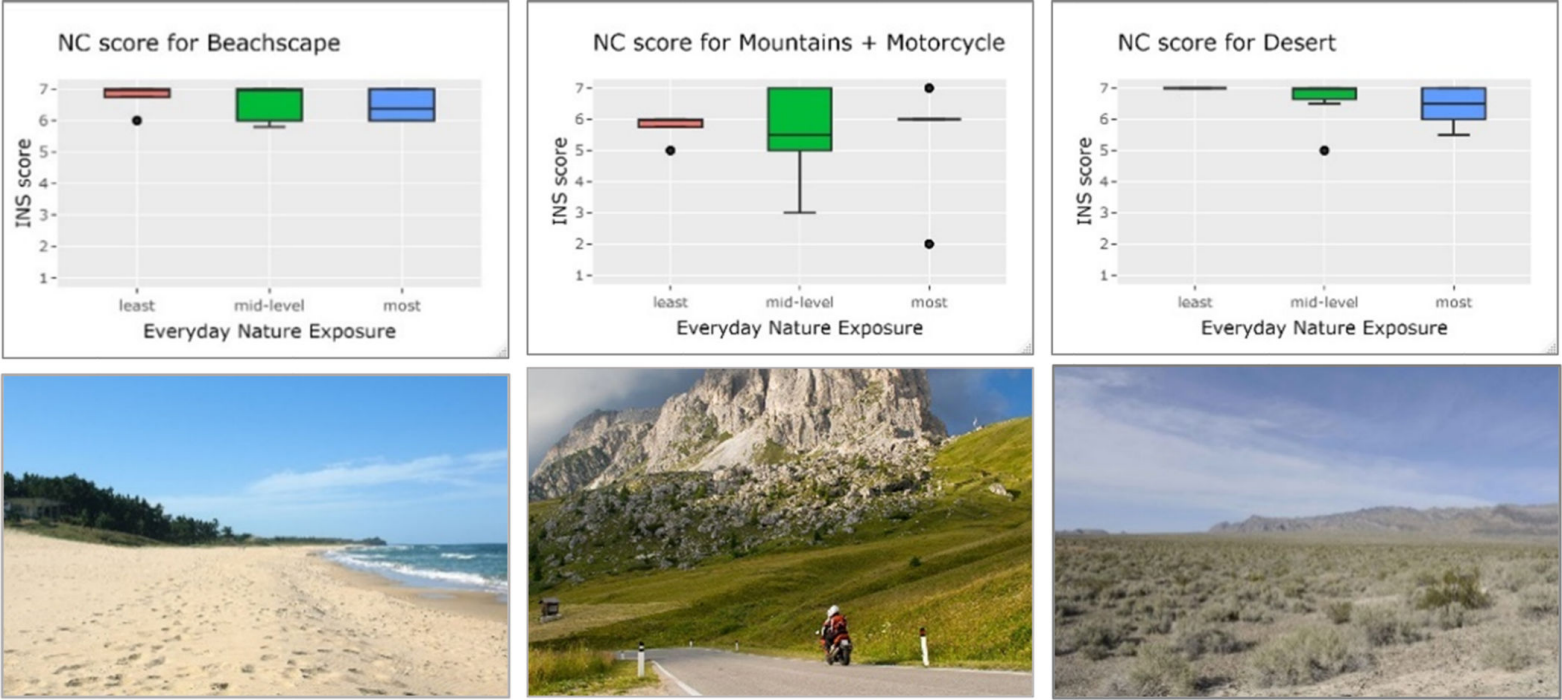

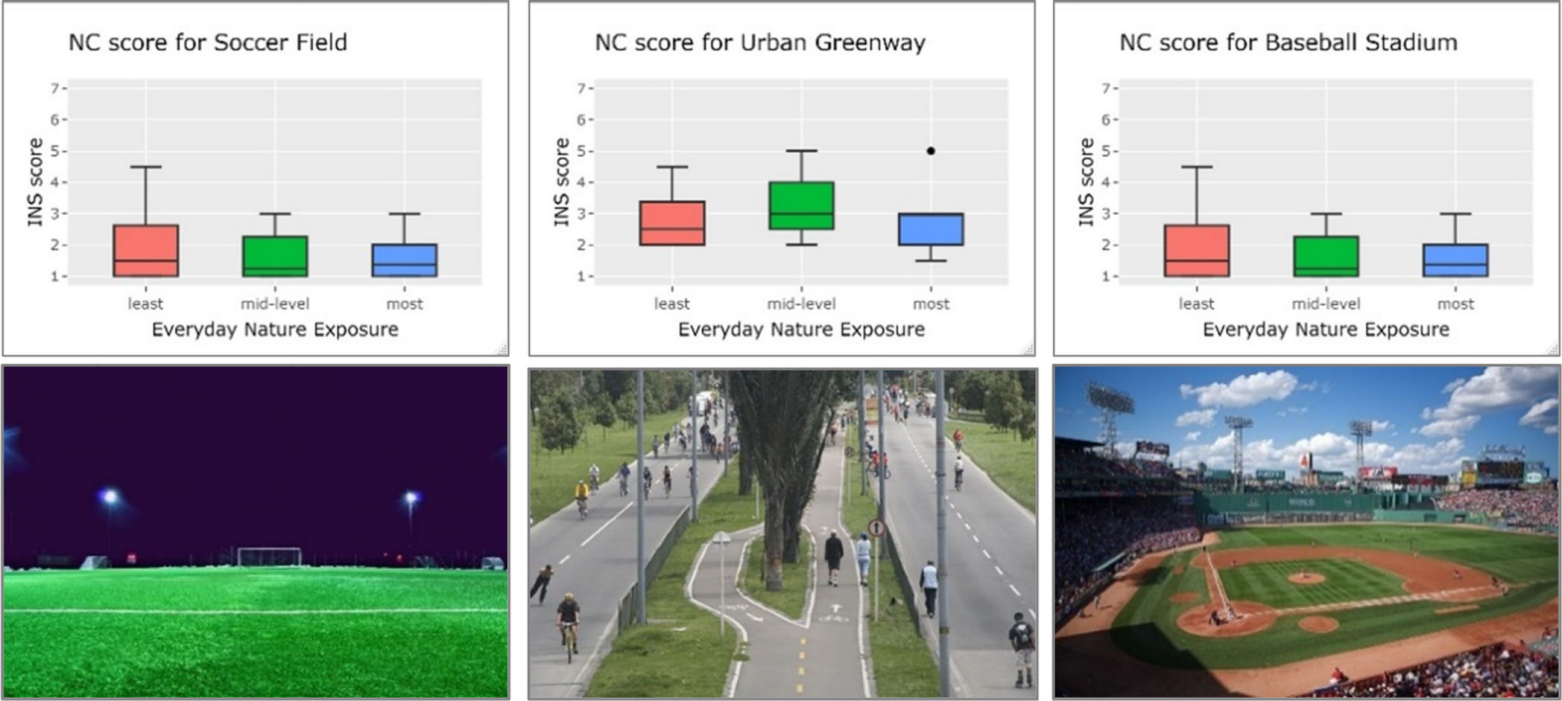

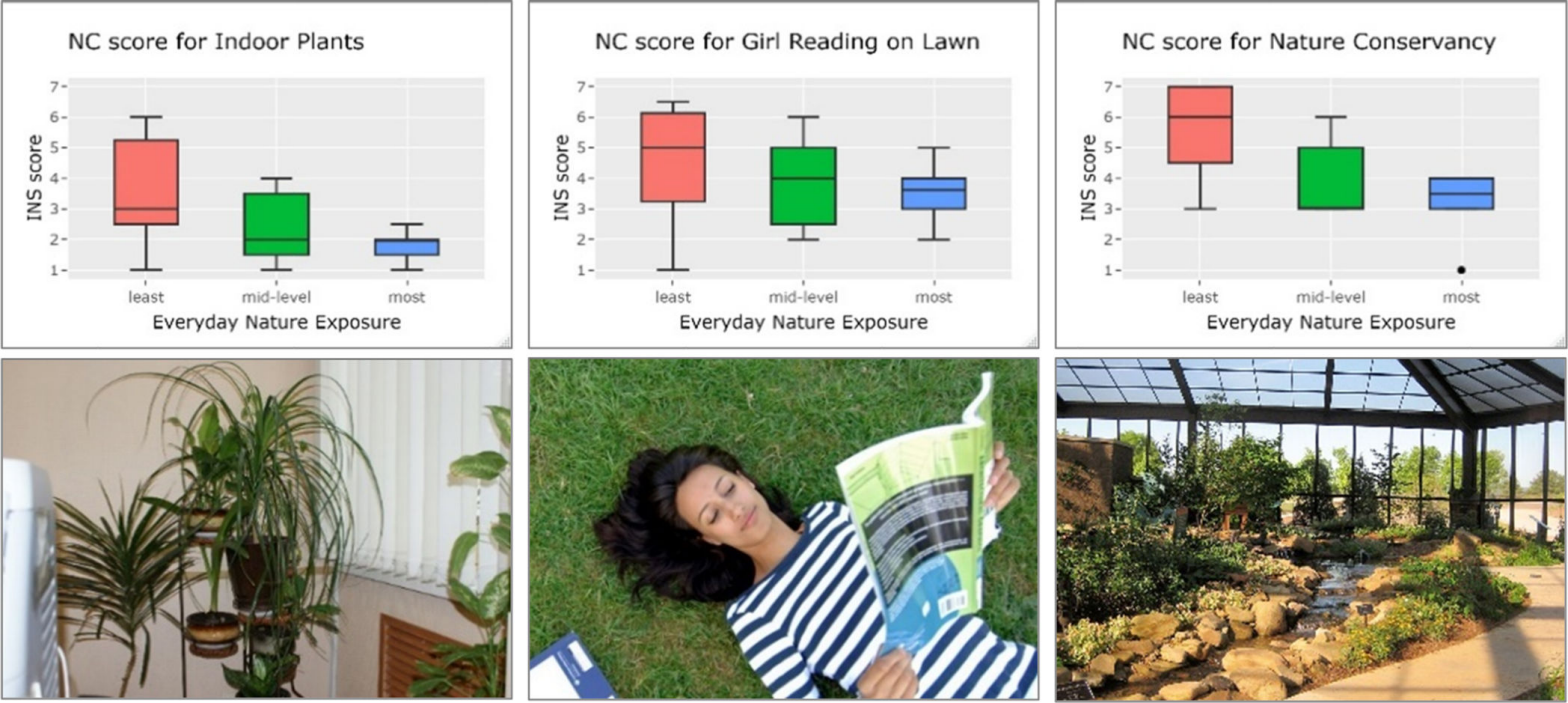
The results of the NC scoring by categorical levels of everyday nature exposure are paired with the type of nature scene. (**a**) Top: All focus groups assigned the highest mean scores of 6–7 to wild nature scenes; (**b**) Center: Urban outdoor scenes received the lowest mean scores of 1–3 from all exposure groups; (**c**) Bottom: Groups exposed to less nature connected to scenes of nature in built environments at consistently higher levels than the medium- or peak-exposure groups.

We had initially hypothesized that individuals with lower standard nature exposure would define nature more liberally, especially nature encountered in settings in built environments. The outcomes of the photo exercise generally met this hypothesis. However, we observed that individuals shed their personal-experience-based definitions of nature in entering the shared emotional terrain of NC for the photo exercise. The overall scoring of the nature images for perceived NC produced surprisingly steady patterns across the categories of group-level nature exposure. The NC scores converged at both the high and low extremes of the seven-point INS scale, indicating groups’ agreement on nature contexts that did and did not elicit nature affinity. Images of wild nature uniformly received high INS scores, while the low scores that were assigned to scenes of outdoor recreational facilities, including an urban greenway, expressed generalized disconnection from nature (Figure 4).

Categorizing the focus groups by everyday nature exposure did not generate linear mean scoring trends or predictably lower standard deviations (Table S1). However, the photo subgroups representing the lowest and highest everyday nature exposure bookended the rankings of the INS means (SD) among all subgroups [5.87(1.16); 3.24 (2.37)], which was reflective of the aggregate findings determined by the categorical exposure levels: low—4.43 (2.06); medium—4.25 (1.97); peak—3.92 (2.00), on a seven-point scale. Low-nature-exposure groups viewed and evaluated scenes of nature in the built environment more favorably than those living and working in nature-rich environments. We removed two images, *“Leave no Trace”* and *“Strava read-out,*” which evoked nature non-pictorially and whose symbolism was unfamiliar to individuals in the low-exposure groups. The removal of these images resulted in the determination of significant NC differences by the Kruskal–Wallis test as measured by the group-level nature exposure (Kruskal–Wallis χ2 = 6.3445, df = 2, *p* = 0.042). A pairwise comparison showed significance between the groups with the lowest and highest levels of nature exposure (*p* = 0.047) (Table 5).

## 4. Discussion

### 4.1. Concurrence and Disagreement in the Findings

From the individual and group conceptualizations of nature, we discerned three distinct prototypical classifications of how place, familiarity, and experience shape opinions of what constitutes ‘nature’. The semi-structured discussion content differed from the NC scoring of nature-based scenes in some ways. The consensus process for the photo exercise provided the research team with the opportunity to examine vocalized reasons for complementarity and disagreement between the two data collection methods that individual scoring methods would not have revealed. Visual cues contained in the nature photos often reified participants’ comments about nature attitudes that were voiced earlier [147]. As compared to abstracted nature concepts, the inclusion of pictorial images furnished “anchors of meaning” that focused on the contingent process of meaning-making around nature [118]. The range of photos also probed participants’ treatment of the outdoors as a concept distinguishable from nature and how interaction with familiar quasi-natured outdoor settings influenced the NC responses. Most groups did distinguish nature from the outdoors under both methods, further contextualizing each concept according to the specific setting.

Natural vegetation—not parks or outdoor recreational venues—appeared to trigger NC among all groups. This observation suggests inherent biophilic connections, which is consistent with the existing literature on biophilia, which creates propensities to seek out nature as its own end [60,148,149]. These results should inform planning of inclusive outdoor spaces to appeal to the biophilic tendencies underlying many psychological health responses to nature. Further research may investigate how nature-based interventions in urban parks, e.g., sensory gardens, would compare with enhancements to park amenities in, first, enticing individuals into nature and, second, comparing affective changes that follow nature vs. outdoor contact. Knowing how these outcomes differ can inform the design of schoolyards, parks, and community centers for children, adults, and seniors, respectively, who depend on municipal infrastructure for their contact with nature. The latent aspects of contact with nature discussed here—inter alia, wildness and biophilic appeal—can be incorporated into public infrastructure planning to make health and wellbeing explicit as a means of reducing social disadvantage [24,122] through the use of natural systems and processes in designing built environments [150]. If we choose to accept nature contact as a “universal primary need, not a cultural amenity” [151], biophilic design can mediate this needed interaction with otherwise low-natured environments [152–154].

Discrepant NC scores were most apparent in scenes depicting nature in built environment settings. “We discovered that people’s personal experience is divided as a group as far as the artificially created nature vs. those that were wild or unconstructed (not designed). My personal preferences made me hesitate about feeling a connection to nature prima facie” (Female, mid-40s, Berkeley). Urban low-nature-exposure groups recorded higher mean NC and full acceptance of nature in built environment contexts. The stronger affinity for scenes such as “Suburban development”, “Room with plants”, and “Golf course” among these groups may reflect their greater sensitivity to vegetation in urban environments where urban ecology is low. The presence of several teachers in the medium-exposure groups perhaps influenced these groups’ higher NC responses to figurative nature associations, e.g., “Indoor classroom” and “Dandelion in Sidewalk.” Habituation to nature-rich environments among the high-exposure individuals may explain their rejection of urban-nature-based scenes, except as non-natured agents meant to bring people outdoors. The medium- and high-exposure groups debated the tolerance of—and annoyance with—man-made or artificial elements in otherwise natural settings. “The artificiality of nature was definitely something we were divided on” (female, late 30s, USG conservationist). Comments from high-nature-exposure individuals supported prior findings that the presence of the artificial in nature impedes stress reduction [155], leaving open opportunities to investigate stress responses to nature among underexposed populations. The low-nature-exposure groups, who also represented the youngest and lowest SES strata, were the most vocal in discussing environmental degradation and climate change. These groups, more than the medium- and high-exposure groups, connected readily to scenes associated with conservation and environmental education, e.g., “Riverbank clean-up”, “Nature conservancy”, and “Outdoor classroom.” Previous research demonstrated that concern for nature and social justice dovetails with climate change activism among youth and young environmentalists [156–158]. The low-exposure groups also highly scored multisensorial images of urban nature, e.g., “Lawn with girl reading” and “Plaza fountain with children”, emphasizing the subjects’ haptic connection with nature: “The fact that she’s in the open air on a lawn, instead of the library, that is a literal connection of touching nature since she’s laying in the grass” (female, mid-20s, Colombia) and “Her whole head is in the grass, connecting physically with nature” (female, college age, urban Atlanta). Conversely, an individual in the high-exposure group rejected the scenario’s very likelihood altogether: “Sitting in a city context, I feel like this is not something you would normally see in a city” (female, later 20s, Tempe).

Our study revealed several other findings besides those already present in the literature. First, all participant groups, regardless of their nature exposure, responded with equal connection to wild nature scenes and equal disconnection to scenes of low nature content. Such consistent response patterning suggests an intuitive biophilic response to what is and is not nature [47,159] that is rooted in a genetic human propensity toward nature [160], not just in first-hand experiences in nature. Two recent literature reviews independently concluded that humans have a “fundamental psychological need” for NR [51,52], with empirical evidence supporting NC as a “primitive belief” [161].

Second, the dissatisfaction with being outdoors versus in wild nature first expressed in the focus groups was validated by the low NC scores assigned to open-air “artificial” recreation sites in the photo exercise. Urban low-nature-exposure individuals described their nature experiences during the focus group discussions as looking at the sky or playing after-school football, yet did not connect with these same elements when they envisioned themselves as spectators. In fact, photos of outdoor facilities instilled NC only when participants placed themselves as actors in the scenes, e.g., *“Campsite”, “Winter Snowmobiling”,* and *“The Fens”,* rather than as on-lookers, e.g., in *“Plaza fountain with children”* or *“Turfgrass.”* Surprisingly, all three groups responded tepidly (mean INS score = 3.08, SD = 1.10) to *“Urban Greenway”,* with its combined green exercise and amenities offerings. These findings suggest that urban greenspace must invite interactions by city residents in order to be usable for purposes of nature contact and not just plentiful as an NDVI measurement.

Third, the higher INS scores for built environment settings given the by low- and medium-exposure groups but not the high-exposure group signaled that context saliently matters when evaluating nature. Photos that showed natural elements “unnaturally” introduced into artificial or built settings, e.g., *“Lobby green wall”* and *“Urban Playground”,* failed to connect any group to nature. However, the low-exposure groups assigned higher NC scores to scenes of built environments where nature plausibly enhanced structural urban drabness, e.g., *“Outdoor classroom”* and *“Farmers’ market”.* Individuals may be instinctively primed to recognize appropriate translations of biophilic properties but reject others that are less well-suited.

Taken together, these findings support the idea of different conceptualizations of nature among prototypical exposure groups and the meanings that nature use holds for them. The mixed methods used in this study suggest that cultural sets, which include cumulative nature experiences, shape one’s definition of nature, but not one’s intuitive relatedness to nature. Eclectic approaches such as those used here can often expand data analyses to reveal overlooked pathways toward positive nature engagement. Our use of photo imagery as an analytical tool to validate qualitative discussion commentary indicated where along the spectrum of built and natural environments perceptual differences emerged. This was particularly true when the principal conduit of nature familiarity for many city dwellers was the urban setting. The deployment of urban ecology and biophilic design may be useful for car-less urban dwellers for whom natural environments outside expanding city limits have become increasingly inaccessible. At the same time, programs that promote nature experiences of a larger scale and duration and that are free of the visual distractions of artificial built features, which are associated with stress, may appeal to those with low everyday nature exposure if appropriate introductions are made available.

### 4.2. Implications of Findings

The results of this process ought to influence future nature and health studies to consider more nuanced interpretations of how individual participants respond to experiences in nature. To the expanding criteria set for measuring nature contact in nature–health research, we might add “meaning”, i.e., what does nature contact, statically measured, mean for individuals with varying nature relationships within a specific context? Alternatively, if we continue to assess nature–health as a binary exposure–outcome relationship, then cultural sets, which include prototypical exposure, formative introductions, and relatedness to nature, could be added to specify characteristics of those in our study population.

Our findings suggest that a nature construct as expressed by our participants appears bounded by previous nature-based experiences, degrees of contact with biophilic quality, and circumscribed expectations. This research indicates that individuals with low- and high-nature backgrounds regard nature differently when lower-exposure individuals are both urban and of a low SES. Urban residents who are not of a low SES tend to seek out larger landscapes for recreation and, therefore, did not respond with the same affinity to nature found in built environments as did individuals living in nature-poor neighborhoods. The more animated expressions of NC for lower thresholds of urban vegetation among the low-exposure groups may signal an unmet biophilic need stemming from low nature supply rather than from viewing nature differently. Future studies might examine the effects of nature contact among individuals expressing different levels of demand for nature given fixed vegetation supplies. Nonetheless, how individuals conceptualize nature may be less important than their intrinsic biophilic affinity toward natured spaces that are both familiar and unfamiliar.

### 4.3. Study Strengths and Limitations

The participants’ self-selection into the four focus groups that were recruited through Facebook and further scheduling availability likely conditioned the generalizability of the results, and we were not able to reach some desired demographic groups. Young adults of color mostly represented the low SES stratum, so we failed to capture inter-generational diversity in our sampling. The 34 nature images selected for the photo exercise may have missed some inherent conceptual meanings. The process of assigning nature scores by group rather than individually implies that some respondents’ opinions may have been swayed by or lost to group dynamics. The advantage of using photos as proxies of various nature-based settings was muted by not capturing direct physical presence in nature to probe the biophilic effect that underlies nature connectivity.

However, our study’s strengths of design and representation provided for multiple cultural perspectives that are uncommon even in qualitative research. The use of two rather than a single participatory research tool—focus group discussions operationalized with photo imagery—allowed for comparison of quantitative and qualitative results in order to identify and interpret divergent findings. While we are not exhaustively explaining the phenomenon of nature-seeking, we elicit a good description of the diversity of experiencing nature. The study’s design allowed us to analyze representative observations by the sampled groups, and the semi-structured format elicited extensive qualitative data, which is not possible with survey instruments of the requisite brevity.

## 5. Conclusions

This study investigated how individuals with diverse sociodemographic attributes conceptualize and connect with nature. Our preliminarily findings projected a baseline biophilic affinity for all individuals for what is and is not nature overlain by circumstantial responses that were closely aligned with built environment settings. This research also suggests it is not enough to know that sociodemographics predispose individuals to regard nature differently, but it is also necessary to know the contexts and ways in which these differences surface so as to shape opportunities for interacting with nature as a prospect for the enhancement of health and wellbeing. Perhaps it is not necessary to define nature as much as to capture autonomic responses to naturalistic impulses when translating research into practice. Interpretations of what is and is not “known” to be nature can be impartially organized by experiences, locations, and cultural sets to sharpen nature exposure constructs in order to assess their associated health outcomes.

## Supplementary Material

Fig S1_Focus group locations

Table S1_Focus group characteristics

Table S2_Topic guide for focus groups

Fig_S2_Photos

## Figures and Tables

**Table 1.** Categorization of focus groups according to the level of everyday nature exposure.

Nature exposure	Focus Group Site	Group Characteristic	Age Range	N=	Comment

High	Rural WV	Government conservation agents	40–65	15	*I was able to get a seasonal conservation law enforcement position with the state. And it was my first introduction to the environment. And I’m like, wait a minute, just like so many guys— click— I can bring both these passions together and this is my future.*
High	Suburban CT	Land trust members	50+	19	*I’m definitely one of those introverts who likes to go into nature to renew and get away from people. So if iťs nice weekend weather, I may not go because I know that I am not going to get that nice peaceful feeling that I would like. So having an area thaťs not overly populated is really helpful.*
High	Tempe, AZ	Graduate Students	25–35	11	*I never consider any sort of city or like urban, I almost never consider anything like that to be nature because iťs all been sculpted. Even if there are trees and stuff around, they’ve almost certainly been planted there by human hands and the soil thaťs there is not a native soil for that type of plant species*
Medium	Suburban Atlanta	Facebook ad respondents	25–80	11	*So I guess it would be -for me to really feel like I’m in nature - I would not want sounds of the man-made environment.*
Medium	Berkeley, CA	Facebook ad respondents	25–70	13	*Right now where I go for nature is the Bay’s regional park. Amazing they’re so close. It was the city right up to the edge and then forever green. Built-up since we moved here. But there’s still park forever. I think I really am drawn to urban living and wilderness longing.*
Medium	Boston, MA	Facebook ad respondents	25–75	14	*I feel like my definition of nature has changed depending on where I am. Out west, I would not consider an arboretum an example of nature but in Boston I would. It also depends on the density of the population. So inside a city where there’s so many people, I would consider a patch of green free of noises and stuff to be part of nature.*
Medium	Suburban Phoenix	Facebook ad respondents	40–70	10	*The takeaway is that the more familiar you are with the processes of nature, the more restricted it is how you define it, with the definition, with its limits. The more restricted maybe i is how, what you define it as where it limits or changes.*
Low	Urban CT	Community youth mentorship program	18–22	10	*I’ve been to other areas, like I was thinking of Louisville, Kentucky which is nice, because I guess I donť like a more rural area, I do like a mixture of I guess more suburbanish, if not urban. But the feeling of being in a place where you’re like, ok - iťs modern but there’s still a sense of, you know, trees.*
Low	Downtown Atlanta	Undergraduate students	18–22	15	*I feel like I want to go to a mountain or something more scenic, zohich is where nature is to me, I have to get in my car and drive at least tzvo hours. So I fed like this city is helping me redefine zvhat nature touchpoints may be and bringing those to me but I donť knozo if iťs making it more effortless.*
Low	Sacramento, CA	Latin American childcare workers	24–28	8	*Today, technology means there is no time to even take a 10-minute walk outside. So everyone gets lazy and reaches for the easiest, most accessible thing there is to do rather than any effort to go outside. When someone is in a new place, or a foreign country, what you want to do most is to explore. I donť have a car, but I take the kids’ stroller and take them to explore.*

**Table 2.** The participants’ comments reflect non-consensual views of a nature construct.

■ *I think nature is so insanely subjective from person to person that it really depends on your definition of nature. Because, for example, I think we all have an idea of a national park being, “nature,” but what else counts?* Female, mid-40s, Berkeley
■ *At least define what nature is, because there’s 20 different definitions of nature right here, right?* Male, late 40s, conservationist
■ *So everybody’s nature thing is different, everybody’s definition of it is different. I don’t knozv, zee do need to change [that perspective].* Female, 18 y/o, Urban CT
■ *My takeazvay is that the more familiar you are with the processes of nature, the more restricted your definition is - its limits, its changes.* Female, early 50s, Berkeley
■ *I think there’s alzuax/s gonna be this spectrum of zvhat nature is, wehether it’s our own little garden that we plant, or that which is the other extreme of being out in the wilderness.* Female, late 30s, Atlanta

**Table 3.** Experience in nature conditions acceptability for what is and is not nature.

■ *I realize now how important one’s experience in nature is in different places at different times and different points in life.* Male, mid-60s, Phoenix
■ *Nature is very subjective due to your own individual experiences. It’s seems like what you agreed on was that my personal prejudices made me hesitant about connecting. It’s not lifestyle, it’s experiential. How you react to the experiences.* Female, late 20s, Berkeley
■ *For me, it would depend if, for example, that little dandelion in the sidewalk, if I really wanted to study that and find the nature that I would, then I can, my instant gratification is no.* Male, mid-50s, conservationist
■ *Flipside is, though, if I’m in a really urban environment, I gravitate to whatever looks like life. So that little dandelion in something that’s really nature. I’m not probably paying much attention to it, but if all I see is concrete, it looks pretty damn good.* Female, mid-40s, conservationist

**Table 4.** Outdoors and nature are not the same concept for most individuals.

■ *I don’t think outdoors and being in nature are the same thing. 1 feel like there’s a difference. Because when I walk to my bus in the morning, I’m outside but I don’t really feel around nature. Because it’s mostly me walking by buildings, houses, I go across the bridge, I look over the side and I see a freeway. And I get on the bus and there’s no really nature around me. But then when I worked at a summer camp there was nature everywhere. There was trees long as I could see, there was hiking trails, there was a nice river. There’s a big difference between both.* Male, college-age, urban CT
■ *I could be outdoors and not feel like I’m in nature. I have dogs, I have to take them outside twice a day, I’m always outside. But I would have to actually be more aware to feel like I’m in nature. So sometimes I’ll look around because there’s a wooded area by my apartment or I’ll notice fireflies at night. That makes me feel like I’m in nature but just being outdoors for me doesn’t feel like I’m in nature, it just feels like outside.* Female, college age, Urban Atlanta
■ *Outdoors is a soccer field. Nature is Mt. Ranier.* Male, early 50s, conservationist
■ *It’s like a continuum. Outdoors. You’re outside. It has some nature. Bugs or dog, whatever. But I don’t think of being in the middle of Phoenix as being in nature. You know, I want to go out even if I’m outdoors. Yeah, I’m outsi where there’s not manmade structures or rivers. And the further away from people and people-things is more nature.* Male, early 60s, Phoenix

**Table 5.** The Kruskal–Wallis rank sum test for a three-way group comparison shows that background levels of everyday nature exposure significantly predicted NC across the photo images. Pairwise tests yielded a significant difference in NC scores between the groups with the lowest vs. highest levels of everyday nature exposure.

	*p*-values	
Kruksal-Wallis rank sum test^1^	0.042	
Kruksal-Wallis pair-wise tests	Low exposure	Medium exposure
Medium nature exposure	0.314	---
High nature exposure	0.047	0.119

1 Kruksal–Wallis tests are assumed to be statistically significant at *p* < 0.05.

## Data Availability

The data are presented in the paper.
