# Supplementary figures and images for: Cultural Sets Shape Adult Conceptualizations and Relationships to Nature
Source: Sustainability. Author manuscript; Available in PMC 2023 Feb 10. (PMC9912744; doi:10.3390/su132011266)

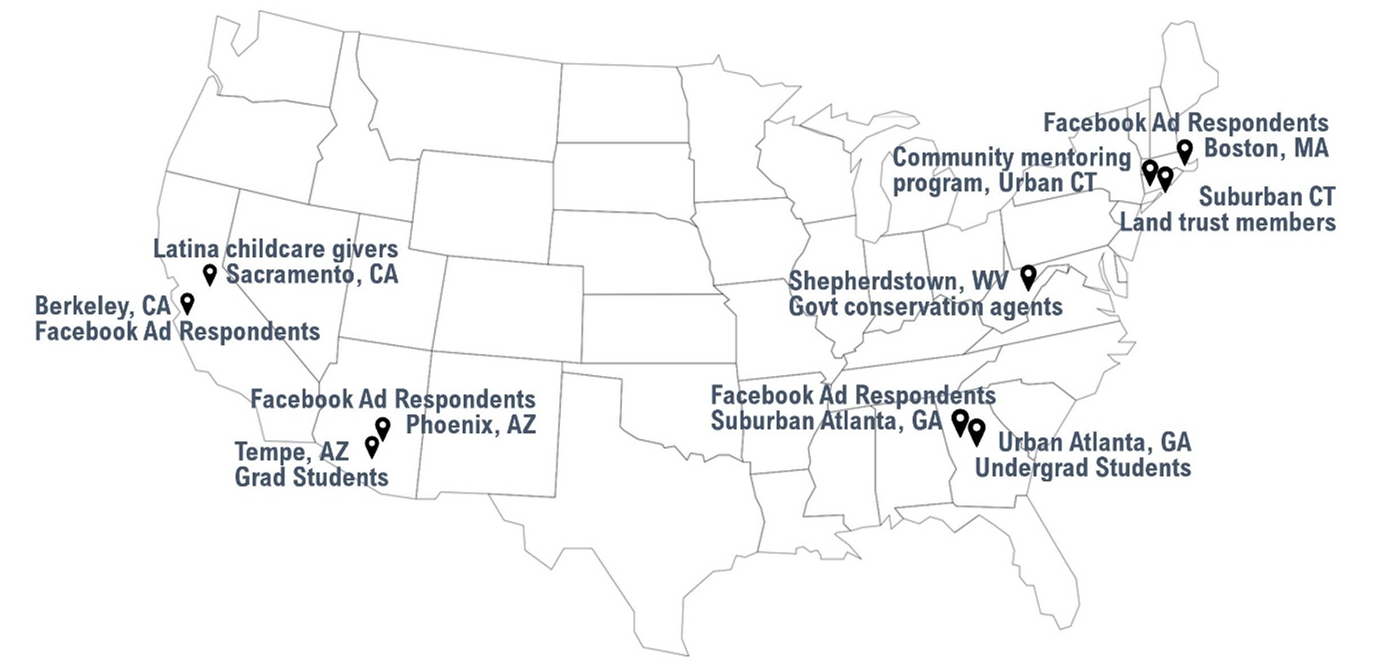

Supplement: Fig S1_Focus group locations [file NIHMS1860995-supplement-Fig_S1_Focus_group_locations.tif]
