# Supplementary material for: Cultural Sets Shape Adult Conceptualizations and Relationships to Nature
Source: Sustainability. Author manuscript; Available in PMC 2023 Feb 10. (PMC9912744; doi:10.3390/su132011266)
Supplement: Table S1_Focus group characteristics [file NIHMS1860995-supplement-Table_S1_Focus_group_characteristics.docx]

Table S1: Focus Group Characteristics

| **Group** | **Location** | **Group Characteristic** | **Gender** | **Age Range** | **# Indiv** |
| --- | --- | --- | --- | --- | --- |
| 1 | Tempe, Arizona | Graduate Students | 6 F, 5 M | 25-35 | 11 |
| 2 | Suburban Phoenix | Phoenix Facebook ad respondents | 4 F, 6 M | 40-70 | 10 |
| 3 | Rural West Virginia | Government conservation agents | 5 F, 10 M | 40-65 | 15 |
| 4 | Boston, Massachusetts | Boston Facebook ad respondents | 11 F, 3 M, 1 NB | Mixed age | 15 |
| 5 | Urban Connecticut | Community mentorship program | 2 F, 8 M | 18-22 | 10 |
| 6 | Downtown Atlanta | Undergraduate students | 13 F, 2 M | 18-22 | 15 |
| 7 | Suburban Atlanta | Atlanta Facebook ad respondents | 9 F, 2 M | Mixed age | 11 |
| 8 | Berkeley, California | Berkeley Facebook ad respondents | 9 F, 3 M, 1 NB | Mixed age | 13 |
| 9 | Sacramento, California | Latin American childcare workers | 7 F, 1 M | 24-28 | 8 |
| 10 | Suburban Connecticut | Land trust members | 11 F, 8 M | Over 50 | 19 |
