# Supplementary material for: Cultural Sets Shape Adult Conceptualizations and Relationships to Nature
Source: Sustainability. Author manuscript; Available in PMC 2023 Feb 10. (PMC9912744; doi:10.3390/su132011266)
Supplement: Table S2_Topic guide for focus groups [file NIHMS1860995-supplement-Table_S2_Topic_guide_for_focus_groups.docx]

**S1 Table. Topic guide for semi-structured focus group discussion.**

| **Opening question** |
| --- |
| Recall a place associated with nature which is meaningful to you. |
| **Follow-up questions** |
| - What do you think of when you hear the word “nature”? |
| - Does a definition of nature include, for example, animals … pets … water … desert? |
| - Is being in nature the same as being outdoors? |
| - Do you recall an experience that led you to view nature in a positive or negative way, and at what age did this occur? |
| - Was there a person or organization who introduced or socialized you to nature? |
| - A large newspaper recently wrote, “Here in our part of the country, where access to nature is “effortless”… How effortless is it for you to access nature if at all, and why or why not? |
| - What are some barriers to nature engagement? |
| - Do you think of nature as safe? Is there a difference between risk and safety when in nature? |
