# Supplementary material for: Cultural Sets Shape Adult Conceptualizations and Relationships to Nature
Source: Sustainability. Author manuscript; Available in PMC 2023 Feb 10. (PMC9912744; doi:10.3390/su132011266)
Supplement: Fig_S2_Photos [file NIHMS1860995-supplement-Fig_S2_Photos.pdf]

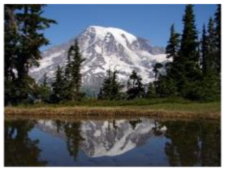
Mt. Ranier

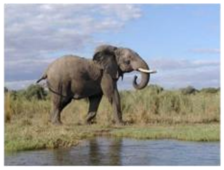
Wild elephant

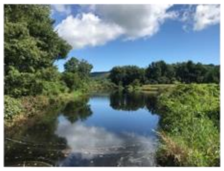
Country Pond

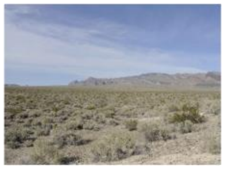
Desert

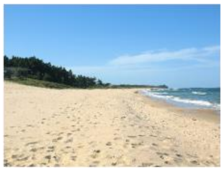
Beachscape

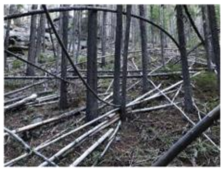
Felled trees

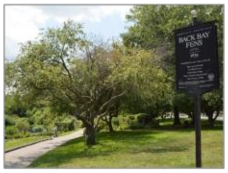
Fens Park

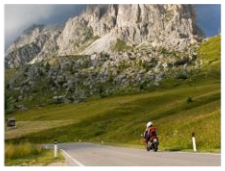
Mountains + Motorcycle

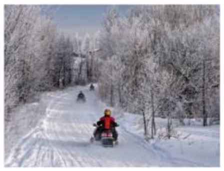
Snowy woods + Skidoo

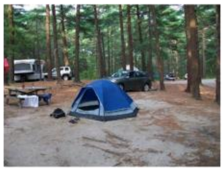
Campsite + cars

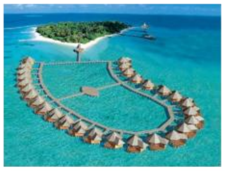
Island + huts

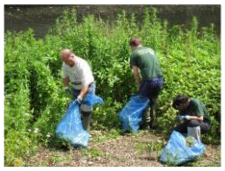
Riverbank clean-up

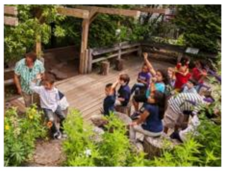
Outdoor Classroom

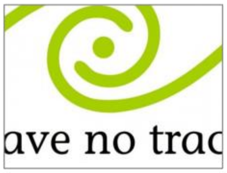

"Leave no trace"

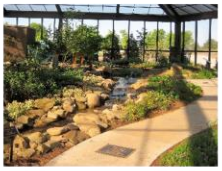
Nature conservancy

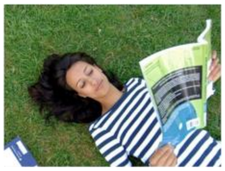
Lawn + girl reading

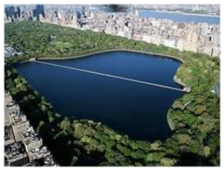
Central Park

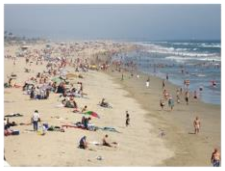
Crowded Beach

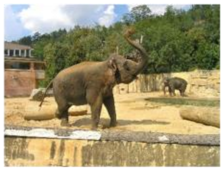
Zoo + elephants

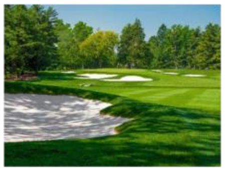
Golf course

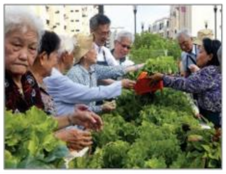
Asian city + market

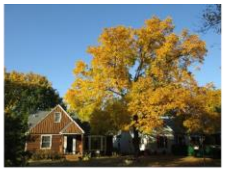
House + maple tree

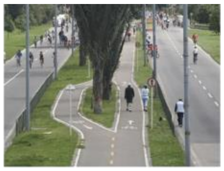
Urban greenway

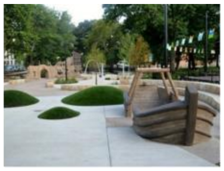
Urban playground

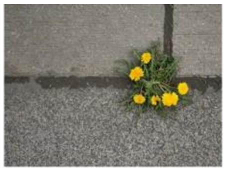
Sidewalk + flower

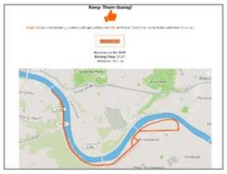
Strava read-out

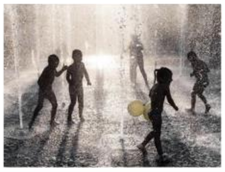
Plaza fountain + kids

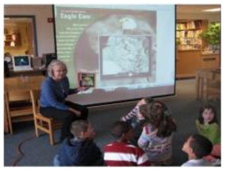
Indoor classroom

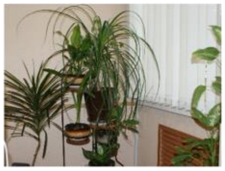
Room + plants

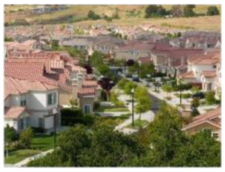
Suburban development

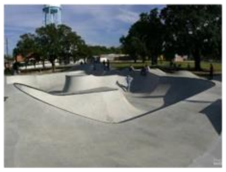
Skateboard park

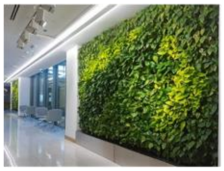
Lobby + green wall

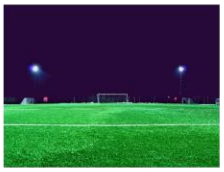
Soccer field

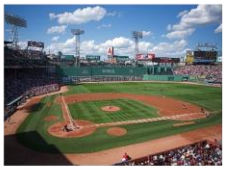
Baseball stadium
